# A novel platelet-rich plasma-filled collagen tube reliably clinically restores meaningful sensory and motor function despite simultaneous long nerve gaps, long repair delays, and increasing age: a case series

**DOI:** 10.3389/fbioe.2026.1900300

**Published:** 2026-10-26

**Authors:** Damien P. Kuffler, Ivan J. Sosa, Onix Reyes, Christian A. Foy

**Affiliations:** 1 Institute of Neurobiology, Medical School, University of Puerto Rico, San Juan, PR, United States; 2 Section of Neurosurgery, Medical School, University of Puerto Rico, San Juan, PR, United States; 3 Doctor’s Center Hospital Manati, Manati, PR, United States; 4 Section of Orthopedic Surgery, Medical School, University of Puerto Rico, San Juan, PR, United States

**Keywords:** autograft, nerve regeneration, nerve trauma, platelet-rich plasma, platelets, PRP

## Abstract

**Introduction:**

Autografts, the clinical “gold standard” technique for restoring function across peripheral nerve gaps, have significant limitations, including sacrificing a sensory nerve and reliably restoring function only for short gaps (<4 cm), short repair delays (<5 months), and young patients (<25). As the value of any of these variables increases, recovery decreases, and when the values of 2–3 variables increase simultaneously, recovery is significantly less.

**Methods:**

This non-blinded, non-controlled case series involved bridging nerve gaps with a novel composition of a never-before-clinically-tested platelet-rich plasma (PRP) within a collagen tube. Before repairing the gaps, the proximal and distal nerve stumps were cut back more extensively than normal to remove potential scar tissue.

**Results:**

Meaningful sensory function was restored to 70% of the nerves, with each nerve developing sensitivity to 4–8 of 8 different types of sensory stimuli, 70% developing fingertip static 2-point discrimination ≤15 mm, and 40% developing meaningful motor function. Meaningful function was restored despite all patients having simultaneously large values of 2–3 of the variables of gap length, repair delay, and age, which normally restrict recovery.

**Conclusion:**

PRP of a novel composition, which the authors recently showed rapidly and reliably eliminates chronic neuropathic pain in all patients, restored meaningful sensory and motor function, despite simultaneously high values of 2–3 of the variables of gap length, repair delay, and age, which normally restrict or prevent recovery. The results show that this technique induces meaningful sensory and motor function, even under very challenging conditions.

## Introduction

Although autografts are the clinical “gold standard” technique for repairing nerve gaps and restoring sensory and motor function (Pan et al., 2020), their efficacy in reliably restoring meaningful sensory and motor function requires sacrificing a sensory nerve and is limited to short nerve gaps, short repair delays, and young patients (Pan et al., 2020; Lans et al., 2023). As the value of any of these three variables increases, the extent of recovery decreases, with recovery limited to none when the values of 2–3 of the variables are simultaneously large (Grinsell and Keating, 2014; Campodonico et al., 2020). These limitations result in <50% of autograft nerve repairs restoring meaningful sensory or motor function (Grinsell and Keating, 2014; Pan et al., 2020). Therefore, most patients with high values for any of the three variables are not offered nerve graft repair surgery and suffer permanent loss of function and chronic neuropathic pain. Therefore, it is critical to develop novel techniques that, without sacrificing a nerve, promote axon regeneration and meaningful recovery under conditions where autografts have limited to no effect. One promising technique is the application of platelet-rich plasma (PRP).

### Research objectives

This formal prospective pilot study tested whether clinically bridging peripheral nerves with PRP of a novel composition within a collagen tube restored meaningful sensory and motor recovery despite long nerve gaps, repair delays, and older patient ages, even when the values of multiple variables were simultaneously large.

## Materials and methods

### Randomization

Non-randomized.

### Blinding of investigators performing the neurological studies

None.

### Research patients

Patients presenting to the Section of Orthopedic Surgery who required a peripheral nerve gap repair and met the study inclusion criteria.

### Inclusion criteria

Patients 18–75 years old with an upper extremity peripheral nerve gap requiring a repair and having the value of one or more of the three variables simultaneously large, defined as gap length ≥4 cm, repair delay ≥5 months, or age ≥25 years.

### Surgery

Traumatic nerve injuries cause scarring proximal and distal to the nerve trauma site, the zone of injury, and this scarred tissue must be removed to allow axon regeneration. Gunshot injuries induce more extensive scarring than sharp cuts and mechanical traumas. Therefore, under full anesthesia and a microscope, the damaged nerve stumps were trimmed with a scalpel a minimum of 4 mm beyond where well-defined nerve fascicles were visible in the proximal nerve stump, and no scarring was seen in the distal stumps. The proximal and distal nerve stumps were inserted 1–2 mm into the collagen tube. No sutures were used.

### Collagen tubes

Before FDA-approved collagen tubes were accessible, they were made from FDA-approved 2 × 8 cm collagen sheets (Veritas, Synovis Life Technologies, St. Paul, MN). The tubes were created by sewing the sheets around the handle of a surgical tool. Tubes created from 8 cm sheets can be used to repair gaps ≤7.6–7.8 cm in length, allowing the proximal and distal nerve stumps to be 1–2 mm inside the PRP-filled collagen tube. For longer gaps, two collagen sheets were sewn end-to-end and then into a tube. Subsequently, NeuroMend collagen tubes (Regenity Biosciences, Oakland, NJ) were used.

### Preparing and injecting platelet-rich plasma

PRP was prepared and injected as previously described (Kuffler et al., 2025). Briefly, 6 cc of PRP were separated from 55 cc of whole blood using the Gravitation Platelet Separation III (GPS III) centrifuge tube (Zimmer Biomet, Warsaw, IN). PRP and thrombin were simultaneously injected into the collagen tube, and within 20 s, the thrombin polymerized the liquid PRP fibrin into a viscoelastic matrix scaffold, and the patient was closed. Figure 1 shows three completed PRP repairs.

**FIGURE 1:**
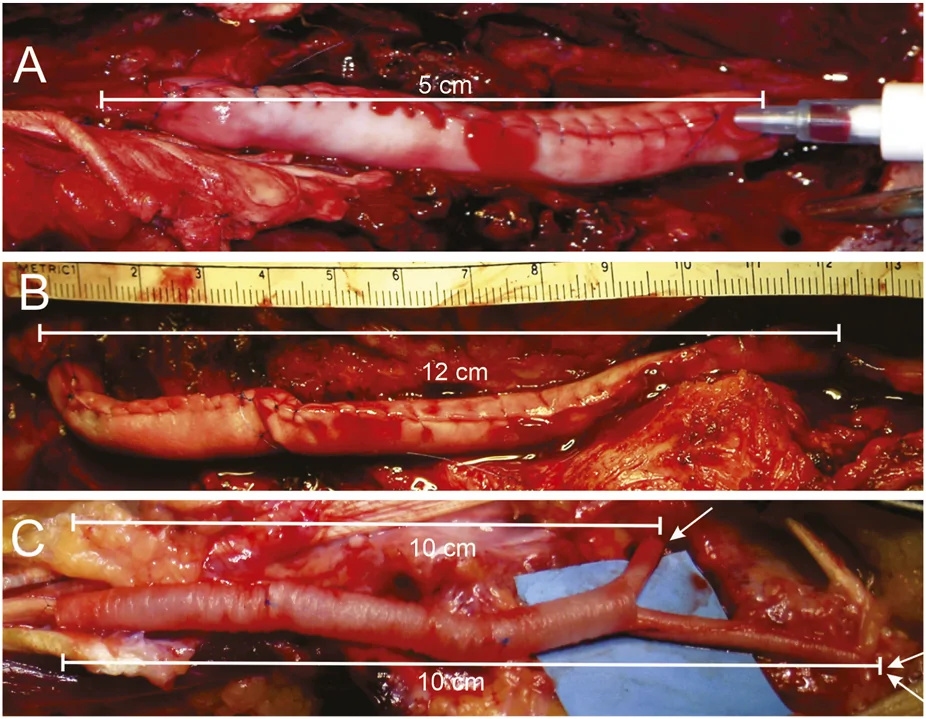
PRP nerve gap repairs. **(A)** Repair of a 5 cm mixed nerve gap with a collagen tube sewn from a sheet of collagen. **(B)** Repair of a 12 cm gap with two collagen sheets sewn end to end. **(C)** Repair of a 10 cm motor nerve gap (tube entry point indicated by a single arrow), and two 10 cm sensory nerve gaps (entry points indicated by two arrows). A Y-ended collagen tube was created by inserting two smaller-diameter NeuroMend tubes into several larger-diameter tubes with overlapping ends.

### Electrodiagnostic studies

Evoked nerve conduction studies (NCS) involved electrically stimulating the repaired nerves proximal to the repair site and recording evoked sensory nerve action potentials with surface or needle electrodes distal to the repair site, and with surface, needle, and monopolar muscle recording electrodes. Electromyographic (EMG) studies evaluated muscle innervation by the repaired nerves, looking for evidence of denervation potentials, active voluntary muscle contraction, and abnormalities in the motor unit potentials that suggest chronic injury and partial or complete recovery. The contralateral extremity nerves were used to compare distal latencies, nerve conduction velocities, and the evoked response amplitudes. A nerve was considered to have axons extending entirely across repaired nerve gaps if any surface, needle, or monopolar muscle recordings were positive.

### Motor recovery

Motor recovery was analyzed using voluntary muscle tests and rated according to the modified British Medical Research Council Score, a 6-grade motor function scale (M0-5), with meaningful motor recovery defined as ≥ M3 (Dvali and Mackinnon, 2003). Depending on the location of the repaired nerve injury, each patient underwent 1 to 4 motor function tests. Each muscle group’s motor force was designated as the mean of the forces generated by the different muscle groups innervated by the repaired nerve.

### Sensory recovery

Sensory testing involved the application of eight different types of sensory stimuli: light touch, pinprick, pressure (using Semmes-Weinstein monofilaments), vibration, heat, cold, proprioception, and static 2-point discrimination (s2PD) using a Disk-Criminator (Sensory Management Services LLC, Lutherville, Md.) with a value of ≤15 mm equal to S3+. The extent of sensory recovery was ranked using the Mackinnon-Dellon sensory recovery scale. Meaningful sensory recovery was defined as ≥S3 (Dvali and Mackinnon, 2003).

### Statistical analysis

Statistical analysis involved performing exploratory multivariable regression with bootstrap confidence intervals analysis.

### Patient follow-up

Due to patient noncompliance with returning for exams and other circumstances beyond the investigators’ control, not all physiological and electrophysiological exams could be performed as planned at 6 and 12 months post-surgery. Therefore, although far from optimal, to ensure consistency in the analysis of the exams, all subjects were brought in and examined by the same clinicians within 2 months. This resulted in recovery times of 10 months–17.8 years, with a mean of 13.3 years.

## Results

### Demographics: patients, nerves, and recoveries

The PRP repairs involved 8 patients, all males, and 10 upper-extremity mixed sensory/motor nerves, with 2–12 cm gaps (mean 6.0 ± 2.6), repair delays of 0.1–3.25 years (mean 1.0 ± 1), aged 18–58 years (mean 36.7 ± 13.7). Table 1. The nerves were radial (11%), median (1.1%), and ulnar (67%), with the injuries located at the wrist (11%), forearm (22%), and elbow (67%). The causes of the injuries were sharp objects (20%), mechanical trauma (30%), and gunshot wounds (50%). Of the patients, 22% had values of 2 variables simultaneously large, 78% had values of 3 variables, and 100% had values of 2 or 3 variables simultaneously large.

**TABLE 1:** Demographics and recoveries.

Demographics/Recoveries	PRP repairs
# Patients	8
Gender	Male
# Nerves repaired	10
Gap lengths	2–12 cm (mean 6.0 SD 2.6)
Repair delays	0.1–3.25 years (mean 1.0 SD 1)
Patient ages	18–58 years (mean 36.7 SD 13.7)
Motor recovery ≥ M3	40%
Sensory recovery ≥ S3	70%
Nerves with sensitivity to 4–8 types of sensory stimuli	100%
2-Point discrimination ≤15 mm	70%
Repairs with values of 2 variables simultaneously large	22%
Repairs with values of 3 variables simultaneously large	78%
Repairs with values of 2–3 variables simultaneously large	100%

### Electrodiagnostic analysis

Electrical stimulation of the PRP-repaired nerves proximal to the repair site evoked sensory nerve action potentials recorded with surface or needle electrodes distal to the nerve repair site from all the repaired nerves. They had prolonged onset latencies that decreased over time, with normal conduction velocities and amplitudes. These results indicate that some axons of each repaired nerve regenerated entirely across the repaired gaps.

Needle EMG of resting reinnervated muscle revealed fibrillations, denervation potentials, and positive sharp waves, indicating abnormal activity of single muscle fibers and absent or reduced voluntary recruitment of motor unit action potentials. Motor unit action potentials had increased phases of the potential, compatible with partial reinnervation. Thus, despite axons reinnervating each denervated muscle, the repaired nerves suffered substantial axonal loss, regenerating entirely across the repaired gaps, resulting in most patients exhibiting incomplete muscle recruitment and motor unit potential morphology compatible with partial reinnervation.

### Motor recovery

Physiological studies showed that despite all nerves having values of 2–3 of variables that normally reduce or block axon regeneration and recovery being simultaneously large, PRP repairs resulted in 40% of the nerves restoring meaningful motor recovery. Motor recovery had a median of M2 (IQR, M0–M4), with 4 of 10 nerve repairs (40%) achieving ≥ M3 motor recovery.

### Sensory recovery

Physiological studies showed that despite all the nerves having the values of 2–3 of variables that reduce or block axon regeneration and recovery being simultaneously large, PRP repairs resulted in 70% of the nerves restoring meaningful sensory recovery of ≥S3. The mean sensory recovery was S3 (IQR, S2–S3+).

Of the repaired nerves, 70% achieved static fingertip two-point discrimination (s2PD) of ≤15 mm, with each nerve, recoveringsensitivity to 4–8 of the 8 different types of applied sensory stimuli. The mean was 7–15 mm (IQR, 7–15 to ≥16 mm, with static 2-point discrimination of 2–6 mm by 10%, 7–15 mm by 60%, and ≥16 mm by 30% of observations.

Each nerve restored sensitivity to 4–8 of the different types of allied sensory modalities, with a mean of 5 (IQR, 3.5 sensory modalities).

## Discussion

Autografts, the clinical “gold standard” technique for restoring function across peripheral nerve gaps, suffer significant limitations. Among these are requiring surgery to remove an autograft, sacrificing a sensory nerve function, and reliably inducing meaningful recovery only across nerve gaps ≤4 cm (Hoben et al., 2018), repair delays ≤5 months (Karabeg et al., 2009), and patients ≤25 (Karabeg et al., 2009). Further, the extent of recovery decreases rapidly as the value of any of these variables increases, with little to no recovery when 2–3 are simultaneously large. The present study tested whether PRP nerve repairs induced meaningful sensory and motor recovery when each patient had the value of at least one variable larger than when autografts are normally effective. It should be noted that the PRP applied in this study to nerves evoking chronic neuropathic pain reliably eliminated the pain in all the patients (Kuffler et al., 2025; Kuffler et al., 2026).

### Rationale for testing PRP efficacy in restoring meaningful sensory and motor function

Although applying PRP to injured nerves in animal models and clinically is reported to promote and enhance the extent of axon regeneration and meaningful recovery, confidence in PRP efficacy has faced several challenges. This is because some studies reported that PRP induced little to no axon regeneration, and there is large variability in PRP efficacy within and between studies.

PRP applied to rat sciatic nerve transection sites was ineffective, especially under suboptimal conditions, suggesting that it does not compensate for poor regenerative conditions (Sariguney et al., 2008). Because clinical nerve injuries rarely occur under ideal conditions, it has been argued that PRP cannot be effective clinically (Sariguney et al., 2008). PRP was ineffective when filling a conduit bridging rat sciatic nerve gaps (Piskin et al., 2009).

Far more studies found PRP to be effective in rats, such as enhancing axon regeneration when applied to nerve injury sites (Anjayani et al., 2014; Liao et al., 2023), neurorrhaphy sites (Sariguney et al., 2008), short nerve gaps (Farrag et al., 2007; Giannessi et al., 2014; Aufiero et al., 2015), when injected into autografts (Teymur et al., 2017; Ikumi et al., 2018), allografts (Zheng et al., 2014), conduits (Hama et al., 2024), vein grafts (Kim et al., 2014), onto sheep nerve crush sites (Sanchez et al., 2017), rabbit cornea (Javaloy et al., 2013), and in rabbits, when injected 14 days after injury (Pandunugrahadi et al., 2022).

Clinically, PRP was effective when applied to the outside of the injured sciatic nerve (Yang et al., 2025), restored function when applied to the peroneal nerve (Sanchez et al., 2014), following intraneural injection (Garcia de Cortazar et al., 2017), when injected onto axons after an epineurotomy (Xie et al., 2025), injected intraperineurially into nerves (Anjayani et al., 2014; Sanchez et al., 2014; Galan et al., 2022), applied to radial nerve primary repair sites following a 4-month repair delay (Garcia de Cortazar et al., 2017), when used to fill a conduit bridging a nerve gap (Wangensteen and Kalliainen, 2010), and applied following carpal tunnel surgery (Malahias et al., 2015). A recent systematic literature review found that for both short and long nerve gaps, autografts restored meaningful sensory and motor function to 71.8% and 56.0% of nerves, respectively (Lans et al., 2023).

For simplicity, most studies examining PRP influence on the restoration of meaningful function were performed on nerves under optimal conditions: following sharp transections, no repair delay, and on young adults. No animal or clinical studies tested the effects of increasing repair-delay-, gap-length-, or age-dependent influences on PRP efficacy when all three variables were simultaneously large. No studies have ever tested the efficacy of this novel PRP.

### Electrodiagnostic studies

Surface electrical stimulation of each nerve proximal to the repair site and using needle electrodes distal to the repair site recorded small compound muscle and sensory action potentials from each nerve. Their prolonged distal latencies and slow motor nerve conduction velocities indicate that, although there was a substantial loss of axons regenerating entirely across the repaired nerve gaps, some axons of each nerve regenerated entirely across the gaps.

Needle EMG recordings from resting reinnervated muscle recorded fibrillations, denervation potentials, and positive sharp waves, indicating abnormal activity of single muscle fibers and reduced or no voluntary recruitment of motor unit action potentials. Motor unit action potentials had increased phases of the potential, compatible with partial reinnervation. Thus, despite axons reaching and reinnervating each denervated muscle, 40% recovered meaningful function, while 60% exhibited incomplete muscle recruitment and motor unit potential morphology compatible with partial reinnervation. These results support the finding that innervation is not well correlated with meaningful recovery (Cannoy et al., 2016; O'Daly et al., 2016).

### PRP of a novel composition promotes meaningful recovery

In this study, PRP repairs resulted in 70% of the nerve restoring meaningful sensory and 40% meaningful motor recovery. All nerves that restored meaningful sensory recovery responded to 4–8 of the 8 applied sensory stimulus types, with 70% restoring ≤15 mm 2-point static discrimination. These recoveries were despite all patients having values of 2–3 of the variables that normally restrict or prevent recovery simultaneously large.

### Influence of increasing gap length, repair delay, and age on sensory and motor recovery following primary nerve, autograft, and PRP repairs

Clinically, under optimal conditions of young patients with no repair delay, PRP applied to primary nerve repairs restored meaningful sensory function to 85%–90% of the nerves and meaningful motor function to 70%–90% (Ruijs et al., 2005). In similar patients, following autograft repairs, recovery was less, with 80%–90% restoring meaningful sensory, and 70%–85% meaningful motor recovery. In animal models, muscle recovery decreased with increasing repair delay, and after 3–6 months’ denervation, muscles’ maximum force capacity decreased to 40%–70% of the original, after 12 months to 10%–30%, and after 18–24 months to 10%–20% (Wu et al., 2013; Carlson, 2014). Similarly, in the present study, the efficacy of PRP was influenced by extrinsic factors, and the involvement of intrinsic factors is suggested.

Clinically, following primary nerve repairs, when the values of 2–3 variables were simultaneously large, 10%–25% of nerves restored S3+ sensitivity, and <5% S4 (Ruijs et al., 2005), with only 5%–15% meaningful motor recovery. Recovery decreased further when the values of all three variables were simultaneously large, with 10%–20% restoring meaningful sensory function and 5%–10% restoring meaningful motor function. However, the novel PRP restored meaningful sensory and motor function even when all the nerves had values of 2–3 of the variables simultaneously large.

This decrease in meaningful recovery is attributed to axotomy causing sensory and motor neurons to lose their capacity to regenerate (Gordon et al., 2011; Vigneswara et al., 2013; Dubovy et al., 2018; Wagstaff et al., 2021; Gordon, 2024; Muhlestein and Mahan, 2026), with sensory neurons retaining a greater intrinsic regenerative capacity than motor neurons. The decrease in meaningful muscle recovery with increasing repair delay is attributed to muscle atrophy and loss of their capacity to generate force (Gordon and Tyreman, 2023). These results provide further support for the well-established recommendation that achieving maximum functional recovery requires minimizing repair delays (Sulaiman et al., 2002; Ramachandran and Midha, 2019).

### Statistical analysis

Exploratory multivariable linear regression analyses examined the associations of nerve gap length, repair delay, and patient age with sensory and motor recovery. Due to the relatively small number of repairs, 8 patients and 10 nerves) available for analysis and three variables of the type of nerve traumas (sharp = 2, mechanical = 3, and gunshot trauma = 5), a multivariable model with 3 predictors could not be reliably estimated, even when supplemented with nonparametric bootstrap confidence intervals (2,000 resamples).

For sensory recovery, the overall model explained approximately 46% of the observed variability (R^2^ = 0.459), although the model was not statistically significant (F = 1.70, P = 0.265). Increasing age showed the strongest negative association with sensory recovery (β = −0.125), while gap length demonstrated a weak positive association (β = 0.080). Repair delay showed a positive coefficient, an unanticipated finding likely attributable to sampling variability and the influence of individual observations rather than a true biological effect.

For motor recovery, the regression model explained approximately 19% of the variability (R^2^ = 0.189). Repair delay (β = −0.406) and age (β = −0.033) both demonstrated negative associations with motor recovery, whereas gap length exhibited only a weak positive coefficient (β = 0.140). None of the regression coefficients were statistically significant, and bootstrap confidence intervals remained wide, reflecting the limited sample size.

Although descriptive analyses suggested differences in mean recovery across trauma etiologies, caution is warranted in interpreting these findings given the limited sample size and substantial overlap in confidence intervals. The broad confidence intervals surrounding the correlation coefficients further indicate considerable uncertainty in the estimated effect sizes. Therefore, the findings should be considered exploratory and hypothesis-generating rather than confirmatory.

### Variability in PRP efficacy and what may underlie this

The efficacy and reliability of PRP in promoting meaningful recovery have frequently been questioned due to variability in the findings between and within studies. The simplest explanation is the use of many different PRP preparation devices (Everts et al., 2006; Jo et al., 2013; Pelletier et al., 2013). These result in PRP with platelet concentrations varying by 10-fold, influenced by centrifugal force, centrifuge acceleration rate, speed, and duration, which influence platelet concentration, the ratio of unactivated to activated platelets, the number and bioactivity of the factors within the PRP releasate, how the platelets are activated, its pH and glucose concentration (Soomekh, 2010; Araki et al., 2012; Davis et al., 2012), and which plasma factors are present and their concentrations, (Li et al., 2011), the presence of leukocytes (Shang et al., 2025), with the PRP delivery method influencing mesenchymal stem cell and erythrocyte concentrations (Araki et al., 2012; Boswell et al., 2012). However, even using the same PRP separation method, PRP’s influence varies depending on delivery duration, the number of times it is applied, and the time between multiple PRP applications.

Other variables affecting PRP include the animal species and age, tissue tested, type and cause of injury, how the thrombin is prepared (Kuo et al., 2010), and the type of thrombin used to activate the platelets (Dadsetan et al., 2001; Araki et al., 2012). Additionally, but never considered, are influences of patient age and health status, diet, differences in the patient’s whole-blood platelet concentration, and their consumption of alcohol, tobacco, medicines, and other products (Dohan Ehrenfest et al., 2009; Kuffler, 2015). Reducing the variability in PRP efficacy requires standardizing PRP preparation and application.

### Influence of different trauma etiologies on the extent of recovery

Regardless of the nerve repair technique, meaningful recovery is best following sharp cuts, followed by mechanical trauma, and gunshot injuries (Secer et al., 2008). Following sharp vs. gunshot trauma, sensory recovery is decreased by 10%–25%, while motor recovery is decreased by 20–40 (Kim et al., 2004; Grinsell and Keating, 2014). This is attributed to gunshot injuries causing not only local nerve trauma, but also indirect heat, shock waves, and cavitation effects (Shields et al., 2023), resulting in scarring extending into the proximal and distal portions of the nerve, which must be removed to prevent restricting or preventing axon regeneration (Zhukauskas et al., 2025). Gunshot traumas cause the longest injury zone, followed by mechanical trauma, and then sharp cuts (Xu et al., 2008; Isaacs, 2013).

In the present study, 50% of the nerve injuries were caused by gunshots, with 56% of them restoring meaningful sensory and motor function, which is greater than anticipated. In the absence of real-time histological analysis of the nerve stumps, in an attempt to remove scar tissue that could not be seen, both nerve stumps were trimmed a minimum of 4 mm beyond where, under visual inspection under a microscope, clear nerve fascicles were seen in the proximal stump, and no scarring was seen in the distal nerve stump.

### Reasons this novel PRP may be more effective than that applied in other studies

The study data prove that platelet-released factors induce extensive axon regeneration and meaningful sensory and motor recovery. The study was not designed to determine the mechanisms by which the unique PRP was more effective in restoring meaningful function than PRP studied by others. However, the authors hypothesize that its greater efficacy is due to how the PRP was prepared and applied. (1) The novel PRP was prepared using the Zimmer Biomet GPS III PRP separation system increases the platelet concentration 9.3-fold, several-fold higher than in most studies. This hypothesis is based on findings in rats (Aufiero et al., 2015) and rabbits (Wang et al., 2023) showing that increasing the PRP platelet concentration 2.5–3.5-fold to 7.5–8.5-fold, respectively (Anitua et al., 2008; Machado et al., 2022), is associated with increased PRP efficacy (Grossen et al., 2022). (2) The leukocyte concentration was increased 5-fold, with evidence showing that leukocyte-rich PRP induces more extensive axon regeneration than leukocyte-poor PRP (Vares et al., 2021). (3) This novel PRP has a higher concentration of bioactive factors, based on the Zimmer Biomet data sheet for the PRP from the GPS III centrifuge tube. (4) The PRP was surrounded by a collagen tube, which, in rats, enhances PRP efficacy (Vares et al., 2021).

### Complications

No patient suffered any post-operative complications, such as infections.

## Conclusion

This small, uncontrolled, non-blinded preliminary clinical case series tested whether clinically bridging nerve gaps with a collagen tube filled with PRP of a novel composition induces meaningful sensory and motor recovery under challenging conditions, where the efficacy of autografts is generally significantly reduced or does not develop. Meaningful sensory function was restored by 70% of the nerves and meaningful motor function by 40%, despite every patient having values of 2–3 of the variables that restrict or prevent recovery simultaneously large. The relatively high percentage of nerves restoring good meaningful recovery following gunshot injury repairs is attributed to far more extensive trimming of the proximal and distal nerve stumps. It is proposed that the percentage of nerves that restored meaningful sensory and motor recovery was reduced by axotomy due to the 1-year mean repair delay reducing the neurons’ capacity to extend long axons, and by prolonged muscle denervation leading to atrophy reducing muscles’ loss of capacity to restore full motor force. The authors previously showed that this novel PRP reliably and rapidly eliminates chronic neuropathic pain. Thus, these results show that this novel PRP rapidly and reliably eliminates chronic neuropathic pain while inducing meaningful sensory and motor recovery by overcoming the intrinsic and extrinsic limitations on axon regeneration and recovery imposed by increasing gap length, repair delay, and patient age. Due to the limitations of this pilot study, the results must be validated in a larger, controlled, blinded study.

### Study limitations

Study limitations include its small size and non-controlled, non-blinded design, which make it impossible to reliably compare the relative efficacy of PRP across different types of nerves, following traumas of different etiologies, and to assess whether PRP efficacy decreases with increasing gap length, repair delay, and patient age. Further, generalization of the electrophysiological results may be limited by the wide range of time intervals between nerve injuries and performance studies.

### Adverse events

No patient suffered any adverse event or untoward medical occurrence, and no suspected or unexpected serious adverse reaction, such as infections, life-threatening events, or otherwise medically important reactions.

## Data Availability

The raw data supporting the conclusions of this article will be made available by the authors, without undue reservation.
